# Supplementary material for: Floquet spin states in OLEDs
Source: Nat Commun. 2021 Jan 19;12:465. doi: 10.1038/s41467-020-20148-6 (PMC7815916; doi:10.1038/s41467-020-20148-6)
Supplement: Supplementary file 1 — Supplementary Information [file 41467_2020_20148_MOESM1_ESM.pdf]

Floquet spin states in OLEDs

# - Supplementary Information -

S. Jamali[1,⊥)], V. V. Mkhitaryan[1,⊥)], H. Malissa[1], A. Nahlawi[1], H. Popli[1], T. Grünbaum[2], S. Bange[2], S. Milster[2], D. Stoltzfus[3], A. E. Leung[4,†], T. A. Darwish[4], P. L. Burn[3], J. M. Lupton[1,2,*)], and C. Boehme[1,*)]

[1]Department of Physics and Astronomy, University of Utah, Salt Lake City, Utah 84112, USA
[2]Institut für Experimentelle und Angewandte Physik, Universität Regensburg, 93053 Regensburg, Germany
[3]Centre for Organic Photonics & Electronics, School of Chemistry and Molecular Biosciences, The University of Queensland, Brisbane QLD 4072, Australia
[4]National Deuteration Facility, Australian Nuclear Science and Technology Organization (ANSTO), Lucas Heights, New South Wales 2234, Australia
[⊥]equally contributing authors
[†]Present address: Scientific Activities Division, European Spallation Source ERIC, Lund 224 84 Sweden
*) corresponding authors: john.lupton@ur.de, boehme@physics.utah.edu

Supplementary Notes

Supplementary Notes 1: Calculation of OLED current change on resonance as a function of B_0 and B_1

Supplementary Note 1.1: Description of magnetic interactions

The singlet-triplet basis is the natural choice for the formulation of the spin-pair dynamics in the presence of recombination. In an applied static magnetic field, $\boldsymbol{B_0} = B_0\hat{\boldsymbol{z}}$, the electron and hole spin-up $|\uparrow\rangle_e$, $|\uparrow\rangle_h$, and spin-down $|\downarrow\rangle_e$, $|\downarrow\rangle_h$ states occupy the Zeeman levels $\pm\frac{1}{2}\hbar\gamma B_0$, where γ is the gyromagnetic ratio of electrons and holes. The singlet-triplet spin-pair states are

$$|T_+\rangle = |\uparrow\rangle_e\,|\uparrow\rangle_h,\;\; |T_-\rangle = |\downarrow\rangle_e\,|\downarrow\rangle_h,$$
$$|T_0\rangle = \frac{1}{\sqrt{2}}(|\uparrow\rangle_e|\downarrow\rangle_h + |\downarrow\rangle_e|\uparrow\rangle_h), \tag{1}$$
$$|S\rangle = \frac{1}{\sqrt{2}}(|\uparrow\rangle_e|\downarrow\rangle_h - |\downarrow\rangle_e|\uparrow\rangle_h).$$

The Zeeman levels of individual spins can be coupled by a resonant microwave excitation. In the language of triplets and singlets, resonant microwave radiation couples the triplet spin-pair levels. At the same time, nuclear spins create random effective hyperfine magnetic fields at the spin sites inducing interconversion between the singlet and triplet spin-pair levels. Characteristic magnitudes of these hyperfine fields, $b_{\mathrm{hf},e}$ and $b_{\mathrm{hf},h}$, are in general different for electrons and holes, and define two distinct characteristic hyperfine frequencies, $\omega_{\mathrm{hf},\mu} = \gamma b_{\mathrm{hf},\mu}$, with $\mu = e, h$. The statistical distribution of local hyperfine frequencies is presumed to be Gaussian, following the distribution

$$\mathcal{N}(\omega_{i,\mu}) = \frac{1}{\sqrt{2\pi}\omega_{\mathrm{hf},\mu}}\exp\left(-\frac{\omega_{i,\mu}^2}{2\omega_{\mathrm{hf},\mu}^2}\right), \qquad \mu = e, h, \tag{2}$$

where $\omega_{i,e}$, $\omega_{i,h}$, $i = x, y, z$, are the Cartesian components of local hyperfine frequencies $\boldsymbol{\omega_e}$, $\boldsymbol{\omega_h}$, distributed isotropically.

In terms of the electron and hole charge-carrier spin operators, S_e^i and S_h^i, $i = x, y, z$, the external and internal static magnetic fields (smf) result in the Hamiltonian

$$H_{\mathrm{smf}} = \omega_0(S_e^z + S_h^z) + \boldsymbol{\omega_e S_e} + \boldsymbol{\omega_h S_h},$$

where $\omega_0 = \gamma B_0$. The interaction with a microwave (mw) field of amplitude $B_1 = 2\omega_1/\gamma$ and frequency ω linearly polarized along $\hat{\boldsymbol{x}}$ is given by the Hamiltonian

$$H_{\mathrm{mw}} = 2\omega_1 \cos \omega t\, (S_e^x + S_h^x).$$

To these interactions we add the spin-exchange and dipolar couplings. We assume an isotropic exchange with the simple Hamiltonian

$$H_{\mathrm{ex}} = J\left(\frac{1}{4} - \boldsymbol{S_e S_h}\right).$$

The dipolar coupling requires a more elaborate description. We have

$$H_{\mathrm{dip}} = \sum_{i,k=x,y,z} d_{ik}\, S_e^i S_h^k, \qquad d_{ik} = \frac{\mu_0 \hbar \gamma^2}{4\pi} \langle \frac{r^2 \delta_{ik} - 3 r_i r_k}{r^5} \rangle,$$

where μ_0 is the magnetic permeability, $\boldsymbol{r}$ is the vector connecting the electron and hole coordinates, and $\langle ... \rangle$ indicates averaging over the electron and hole wavefunctions. In our calculations, we take the average dipolar interaction energy of 25 neV, corresponding to $D = (\mu_0/4\pi)\hbar\gamma^2\langle 1/r^3 \rangle \simeq 2\pi \times 6.05$ MHz[1]. We further adopt a simplified picture where the separation of spins within pairs is the same and has a value rendering the average strength D. Thus, the dipolar tensor of a spin pair depends on the (randomly oriented) unit vector $\hat{\boldsymbol{r}}$ connecting the two spin sites as $d_{ik} = D(\delta_{ik} - 3\hat{r}_i\hat{r}_k)$. Combining all the above interactions, we arrive at the total spin Hamiltonian

$$H(t) = H_0 + H_{\mathrm{mw}}(t), \;\; H_0 = H_{\mathrm{smf}} + H_{\mathrm{ex}} + H_{\mathrm{dip}}, \tag{3}$$

where H_0 is time independent but contains random static interactions. The rationalization of the four eigenstates $|\uparrow\uparrow\rangle = |T_+\rangle$, $|2\rangle = \xi_2|T_0\rangle + \eta_2|S\rangle$, $|3\rangle = \xi_3|T_0\rangle + \eta_3|S\rangle$, and $|\downarrow\downarrow\rangle = |T_-\rangle$ of H_0 in Fig. 2b is described in detail elsewhere[2]. In the following, we show that the half-field resonance features clearly seen in our experiment arise due to the dipolar coupling between the electron and hole spins in a pair. Including the finite exchange interaction in addition allows us to attain a more accurate quantitative agreement between theory and experiment.

Supplementary Note 1.2: Dynamics of a weakly coupled spin-pair ensemble

The spin-density matrix of a spin-pair ensemble, ρ, satisfies the stochastic Liouville equation[2,3],

$$\frac{d\rho(t)}{dt} = i[\rho(t), H(t)] + (G/4)\mathbf{1} + \mathcal{R}_{\mathrm{dr}}\{\rho\} + \mathcal{R}_{\mathrm{sl}}\{\rho\}, \tag{4}$$

where the first term describes the spin dynamics due to the magnetic interactions governed by the time-dependent spin Hamiltonian $H(t)$, G is the spin-pair generation rate, $\mathbf{1}$ the identity operator, $\mathcal{R}_{\mathrm{dr}}$ represents the pair dissociation and recombination, and $\mathcal{R}_{\mathrm{sl}}$ the spin-lattice relaxation processes. The spin-dependent recombination processes are described within the singlet-triplet basis. We assume that the pair dissociation occurs at an equal rate, r_d, from all states of a spin pair, whereas the recombination into triplet (T) and singlet (S) excitons occurs with two different constant rates r_T and r_S. In terms of the matrix elements we have

$$\mathcal{R}_{\mathrm{dr}}\{\rho\}_{\alpha\beta} = -(r_d + r_T)\rho_{\alpha\beta} - (k_r/2)(\delta_{\alpha S} + \delta_{S\beta})\rho_{\alpha\beta}, \tag{5}$$

where $\alpha, \beta = T_+, T_0, T_-, S$ enumerate the singlet-triplet spin-pair states, r_d is the carrier-pair dissociation rate, which is assumed to be the same for all spin-pair states, and $k_r = r_S - r_T$ is the difference of singlet and triplet recombination rates, which in our case is presumed to be positive. The spin-lattice relaxation is taken to be of the form

$$\mathcal{R}_{\mathrm{sl}}\{\rho\}_{\alpha\beta} = -(1/T_{\mathrm{sl}})[\rho_{\alpha\beta} - \delta_{\alpha\beta}\mathrm{tr}(\rho/4)]. \tag{6}$$

The first terms of Supplementary Eqs. 5 and 6 have the same effect and the corresponding rates are naturally incorporated into the single decay constant,

$$w_d = r_d + r_T + 1/T_{\mathrm{sl}} \,. \tag{7}$$

The spin-dependent recombination is efficient if the decay rates are smaller than the hyperfine-induced mixing of the spin states, $w_d, k_r \ll \omega_{\mathrm{hf}}$. Also note that, besides the spin-lattice relaxation, Supplementary Equation 4 implicitly incorporates the T_1 and T_2 processes originating from the random hyperfine fields. This is believed to be the dominant channel of electronic spin decoherence so one can expect that $T_{\mathrm{sl}} \gg T_1, T_2$. In our calculations we assume $T_{\mathrm{sl}} \gg 1/\omega_{\mathrm{hf}}$, ensuring the compatibility of the two previous conditions.

The charge current measured in a continuous-wave EDMR experiment is proportional to the steady-state free-carrier concentration. The spin-dependent contribution of the spin-pair ensemble to this quantity is determined by the time average

$$\frac{1}{\tau_0}\int_0^{\tau_0} r_d \mathrm{tr}\rho(t) dt \,, \tag{8}$$

where τ_0 is the characteristic measurement time. For τ_0 larger than the typical spin-pair decay times, $\tau_0 \gg 1/w_d \,, 1/k_r$, the integral in Supplementary Equation 8 comes from the time domain, where a steady state with the density matrix, $\tilde{\rho}(t)$, is reached. Here, $\tilde{\rho}(t)$ is the steady-state solution of Supplementary Equation 4, time-periodic because of the time-periodicity of the Hamiltonian (see Supplementary Equation 24). It is then easy to see that, for τ_0 much larger than the time period of the Hamiltonian, the average Supplementary Equation 8 converges to $r_d \mathrm{tr}\tilde{\rho}_0$, where $\tilde{\rho}_0$ is the static component of $\tilde{\rho}(t)$. Consequently, the spin-dependent current is found by averaging $\tilde{\rho}_0$ over the random spatial orientations of the dipolar tensor and the random local hyperfine fields (see Eq. 4 in the main text).

Supplementary Note 1.3: Floquet theory approach to the spin-pair Hamiltonian

To find the spin-pair dynamics governed by the Hamiltonian Supplementary Equation 3 under a strong microwave excitation of $\omega_1 \sim \omega_0$, we employ the standard quantum-mechanical Floquet

theory[4]. We begin with introducing the Floquet *dressed* states $|\alpha, n\rangle$, forming an orthonormal basis in an infinite-dimensional Hilbert space. The Floquet states are enumerated with a pair of indices, where the Greek index runs over the four spin-pair states (i.e. $\alpha = T_+, T_0, T_-, S$ in Supplementary Equation 1) and the Latin index is an integer. Using the Fourier decomposition of the time-periodic Hamiltonian Supplementary Equation 3,

$$H_{\alpha\beta}(t) = \sum_n H_{\alpha\beta}^{(n)} e^{in\omega t}, \tag{9}$$

the time-independent, infinite-dimensional Floquet Hamiltonian H_F is defined in the matrix representation by Eq. 1 of the main text. The operator H_F is Hermitian and possesses real eigenvalues and orthonormal eigenvectors,

$$H_F|\psi_{\alpha,n}\rangle = \varepsilon_{\alpha,n}|\psi_{\alpha,n}\rangle. \tag{10}$$

From the definition Eq. 1 it follows that the Floquet Hamiltonian has a periodic structure. In particular, with a shift of the integer indices by the same integer k, only the diagonal matrix elements are changed (see Eq. 2 in the main text). The secular equation $\det(H_F - \varepsilon\mathbf{1})$, from which $\varepsilon_{\alpha,n}$ are found, is unchanged if ε is replaced by $\varepsilon + k\omega$. Thus, if ε is an eigenvalue, $\varepsilon + k\omega$ is also an eigenvalue, meaning that one can label the eigenvalues as $\varepsilon_{\alpha,n} = \varepsilon_\alpha + n\omega$, where $\varepsilon_\alpha = \varepsilon_{\alpha,0}$ is chosen between 0 and ω. Furthermore, the components of the eigenvectors obey the periodicity relation

$$\langle\alpha, n + k|\psi_{\alpha,m+k}\rangle = \langle\alpha, n|\psi_{\alpha,m}\rangle. \tag{11}$$

Supplementary Note 1.4: Formal solution of the stochastic Liouville equation

In the subsequent analysis of the stochastic Liouville equation Supplementary Equation 4 we exploit the non-Hermitian Hamiltonian

$$\mathcal{H}(t) = H(t) - i(w_d/2)\mathbf{1} - i(k_r/2)\mathrm{P}_S, \tag{12}$$

where $P_S = |S\rangle\langle S|$ is the projection onto the singlet state. The complex Hamiltonian $\mathcal{H}(t)$ incorporates the first and the third operators of Supplementary Equation 4, as well as the first term of the spin-lattice relaxation, Supplementary Equation 6. In terms of the non-Hermitian Hamiltonian, the Liouville equation is rewritten as

$$\frac{d\rho(t)}{dt} = i(\rho\mathcal{H}(t) - \mathcal{H}^{\dagger}(t)\rho) + \frac{1}{4}\left(G + T_{\mathrm{sl}}^{-1}\mathrm{tr}\rho\right)\mathbf{1}. \tag{13}$$

From simple population gain and loss arguments one can see that, under steady-state conditions, the total spin-pair population is restricted by $\mathrm{tr}\rho \lesssim G/4w_d$ (recall that $r_S > r_T$). Thus, for long spin-lattice relaxation times $4T_{\mathrm{sl}}w_d \gg 1$ the $\mathrm{tr}\rho$ term in Supplementary Eqs. 6 and 13 can be regarded as a small correction to the source term. We take advantage of this fact and neglect the term in Supplementary Equation 13. Below, we check numerically that in the parametric domain of interest the effect of this term is insignificant. Note that the effect of the spin-lattice relaxation is still preserved through the first term of Supplementary Equation 6. After dropping the last term of Supplementary Equation 13 we write its formal solution as

$$\rho(t) = U(t, t_0)\rho(t_0)U^{\dagger}(t, t_0) + \frac{G}{4}\int_{t_0}^{t} dt' V(t, t'), \tag{14}$$

where $V(t_1, t_2) = U(t_1, t_2)U^{\dagger}(t_1, t_2)$ is introduced, and the time-evolution operator is defined in terms of the time-ordered exponential

$$U(t_1, t_2) = T\exp\left[-i\int_{t_2}^{t_1} dt'\mathcal{H}(t')\right]. \tag{15}$$

Supplementary Equations 11 and 12 are treated within the quantum-mechanical Floquet theory[4]. The dressed states $|\alpha, n\rangle$ form an orthonormal basis in the infinite-dimensional Hilbert space of the Hermitian Floquet Hamiltonian H_F, Eq. 1. The Floquet counterpart of the non-Hermitian Hamiltonian $\mathcal{H}(t)$, defined by

$$\langle\alpha,n|\mathcal{H}_F|\beta,m\rangle=\langle\alpha,n|H_F|\beta,m\rangle-i(w_d/2)\delta_{\alpha\beta}\delta_{nm}-i(k_r/2)\delta_{\alpha S}\delta_{S\beta}\delta_{nm}, \tag{16}$$

acts in the same Hilbert space. Matrix elements of the time-evolution operator can be written as

$$U_{\alpha\beta}(t_1,t_2)=\sum_n\langle\alpha,n|e^{-i\mathcal{H}_F(t_1-t_2)}|\beta,0\rangle e^{i\omega nt}\,. \tag{17}$$

Because of the non-Hermitian character of $\mathcal{H}_F$, its right- and left-hand eigenvectors are not the Hermitian conjugates of each other and must be considered independently. For the right-hand eigenvectors we use the ket vectors

$$\mathcal{H}_F|\lambda_{\alpha,n}\rangle=\chi_{\alpha,n}|\lambda_{\alpha,n}\rangle, \tag{18}$$

whereas the left-hand eigenvectors are denoted by bra vectors

$$\langle\lambda_{\alpha,n}|\mathcal{H}_F=\chi_{\alpha,n}\,\langle\lambda_{\alpha,n}|. \tag{19}$$

By normalizing the eigenvectors one can form an orthonormal set with respect to the non-Hermitian scalar product, $\langle\lambda_{\alpha,n}|\lambda_{\beta,m}\rangle=\delta_{\alpha\beta}\delta_{nm}$. Taking the Hermitian conjugate of Supplementary Eqs. 18 and 19 we confirm that the right-hand eigenvectors of $\mathcal{H}_F^\dagger$ are the Hermitian conjugates of the left-hand eigenvectors of $\mathcal{H}_F$, and *vice versa*. We have

$$\mathcal{H}_F^\dagger|\bar{\lambda}_{\alpha,n}\rangle=\chi_{\alpha,n}^*|\bar{\lambda}_{\alpha,n}\rangle,\qquad|\bar{\lambda}_{\alpha,n}\rangle=\langle\lambda_{\alpha,n}|^\dagger, \tag{20}$$

where the asterisk indicates the complex conjugate, and

$$\langle\bar{\lambda}_{\alpha,n}|\mathcal{H}_F^\dagger=\chi_{\alpha,n}^*\,\langle\bar{\lambda}_{\alpha,n}|,\qquad\langle\bar{\lambda}_{\alpha,n}|=|\lambda_{\alpha,n}\rangle^\dagger. \tag{21}$$

Note that the above eigenvalues have imaginary parts of a definite sign, since

$$\Im m(\chi_{\alpha,n}) < 0. \tag{22}$$

The periodicity property Supplementary Equation 11 translates into

$$\langle \alpha, n+k | \lambda_{\alpha,m+k} \rangle = \langle \alpha, n | \lambda_{\alpha,m} \rangle, \qquad \langle \lambda_{\alpha,m+k} | \alpha, n+k \rangle = \langle \lambda_{\alpha,m} | \alpha, n \rangle, \tag{23}$$

with similar relations holding for $|\bar{\lambda}_{\alpha,n}\rangle$ and $\langle \bar{\lambda}_{\alpha,n}|$. Using the periodicity properties, the integrand operator of Supplementary Equation 14 is given by

$$V_{\alpha\beta}(t_1, t_2) = \sum_n \left\langle \alpha, n \left| e^{-i\mathcal{H}_F(t_1-t_2)} e^{i\mathcal{H}_F^\dagger(t_1-t_2)} \right| \beta, 0 \right\rangle e^{i\omega n t}. \tag{24}$$

Furthermore, the normalized eigenvectors ensure the partitions of unity, $\sum_{\alpha,n} |\lambda_{\alpha,n}\rangle\langle\lambda_{\alpha,n}| = \mathbf{1}$, and $\sum_{\alpha,n} |\bar{\lambda}_{\alpha,n}\rangle\langle\bar{\lambda}_{\alpha,n}| = \mathbf{1}$. Using these properties together with Supplementary Eqs. 18-21, we rewrite Supplementary Equation 24 as

$$V_{\alpha\beta}(t_1, t_2) = \sum_{n,\nu,k,\mu,p} \langle \alpha, n | \lambda_{\nu,k} \rangle \langle \lambda_{\nu,k} | \bar{\lambda}_{\mu,p} \rangle \langle \bar{\lambda}_{\mu,p} | \beta, 0 \rangle e^{-i(\chi_{\nu,k} - \chi_{\mu,p}^*)(t_1-t_2)} e^{i\omega n t_1}. \tag{25}$$

The integral in the solution Supplementary Equation 14 can now be taken using the above relation. We find

$$\int_{t_0}^{t} dt' V_{\alpha\beta}(t, t') = \sum_{n,\nu,k,\mu,p} \langle \alpha, n | \lambda_{\nu,k} \rangle \langle \lambda_{\nu,k} | \bar{\lambda}_{\mu,p} \rangle \langle \bar{\lambda}_{\mu,p} | \beta, 0 \rangle \frac{1 - e^{-i(\chi_{\nu,k} - \chi_{\mu,p}^*)(t-t_0)}}{i(\chi_{\nu,k} - \chi_{\mu,p}^*)} e^{i\omega n t}. \tag{26}$$

We note that the first term in Supplementary Equation 14 represents a transient contribution to $\rho(t)$, vanishing after a time interval $(t - t_0) \gtrsim \max(1/w_d, 1/k_r)$, so that it does not contribute to the steady-state density matrix $\tilde{\rho}(t)$ which we are looking for. Moreover, due to the negative imaginary parts of the eigenvalues, Supplementary Equation 22, the term $\exp[-i(\chi_{\nu,k} - \chi_{\mu,p}^*)(t - t_0)]$ in Supplementary Equation 26 decays exponentially for time intervals of the same

order and therefore does not contribute to $\tilde{\rho}(t)$ either. Thus, we arrive at the steady-state density matrix

$$\tilde{\rho}_{\alpha\beta}(t) = \frac{G}{4} \sum_{n,\nu,k,\mu,p} \frac{\langle \alpha, n | \lambda_{\nu,k} \rangle \langle \lambda_{\nu,k} | \bar{\lambda}_{\mu,p} \rangle \langle \bar{\lambda}_{\mu,p} | \beta, 0 \rangle}{i(\chi_{\nu,k} - \chi^*_{\mu,p})} e^{i\omega n t}. \quad (27)$$

Supplementary Equation 27 is the Fourier decomposition of the steady-state density matrix $\tilde{\rho}(t)$, which is periodic with the period of the drive field. In Supplementary Equation 8, which defines the contribution to the spin-dependent current I, the oscillatory components of $\tilde{\rho}(t)$ average to zero as the integration time is large, $\tau_0 \gg 2\pi/\omega$. Thus, the only contribution to I comes from the time-independent component,

$$\tilde{\rho}_{0,\alpha\beta} = \frac{G}{4} \sum_{\nu,k,\mu,p} \frac{\langle \alpha, 0 | \lambda_{\nu,k} \rangle \langle \lambda_{\nu,k} | \bar{\lambda}_{\mu,p} \rangle \langle \bar{\lambda}_{\mu,p} | \beta, 0 \rangle}{i(\chi_{\nu,k} - \chi^*_{\mu,p})}. \quad (28)$$

Using this relation with the periodicity properties, Supplementary Equation 23, we arrive at

$$\mathrm{tr}\tilde{\rho}_0 = \frac{G}{4} \sum_{\alpha,\nu,k} \frac{\langle \lambda_{\nu,k} | \bar{\lambda}_{\alpha,0} \rangle \langle \bar{\lambda}_{\alpha,0} | \lambda_{\nu,k} \rangle}{i(\chi_{\nu,k} - \chi^*_{\alpha,0})}. \quad (29)$$

We find the eigenvalues of $\mathcal{H}_F$ and $\mathcal{H}_F^\dagger$ perturbatively with respect to small k_r, by splitting these non-Hermitian operators into the unperturbed parts equivalent to the Hermitian Floquet Hamiltonian H_F and the perturbation parts $\pm i(k_r/2)\Pi_S$:

$$\mathcal{H}_F = H_F - i(k_r/2)\Pi_S - i(w_d/2)\mathbf{1}, \qquad \mathcal{H}_F^\dagger = H_F + i(k_r/2)\Pi_S + i(w_d/2)\mathbf{1} \quad (30)$$

where $\Pi_S = \sum_n |S, n\rangle\langle S, n|$ is the projection operator onto the dressed singlet subspace. Using quantum-mechanical perturbation theory, we find

$$\chi_{\alpha,n} \approx \varepsilon_{\alpha,n} - i(k_r/2)\langle \psi_{\alpha,n} | \Pi_S | \psi_{\alpha,n} \rangle - i(w_d/2), \quad (31)$$

where $|\psi_{\alpha,n}\rangle$ and $\varepsilon_{\alpha,n}$ are the eigenvectors and eigenvalues of H_F, Supplementary Equation 10. We further note that $\langle\lambda_{\nu,k}|\bar{\lambda}_{\mu,p}\rangle = \delta_{\nu\mu}\delta_{kp} + \mathcal{O}(k_r)$, so the sum in Supplementary Equation 29 is dominated by terms with $\nu = \alpha$ and $k = 0$. To the leading order, the numerators of these terms can be replaced by 1. We keep only these terms to arrive at the expression Eq. 3 in the main text. The approximation leading from the exact relation Supplementary Equation 29 to the perturbative result Eq. 3 is valid when the level spacing of any two stationary states $|\psi_{\alpha,n}\rangle$ and $|\psi_{\beta,m}\rangle$, coupled by the Floquet projection operator Π_S (i.e., $\langle\psi_{\beta,m}|\Pi_S|\psi_{\alpha,n}\rangle \neq 0$), is larger than k_r,

$$|\varepsilon_{\alpha,n} - \varepsilon_{\beta,m}| \gg k_r. \tag{32}$$

This condition appears to be violated under resonance when $\varepsilon_{\alpha,n} \approx \varepsilon_{\beta,m}$. However, a more detailed analysis shows that level anti-crossing occurs due to the finite dipolar and exchange coupling between spins within the pairs, so that $|\varepsilon_{\alpha,n} - \varepsilon_{\beta,m}| \sim |D|, |J|$. Hence, the condition Supplementary Equation 32 is satisfied, and the perturbative result Eq. 3 is accurate over the entire parametric domain, provided that $k_r \ll |D|, |J|$.

Supplementary Note 1.5: Numerical evaluation of the spin-dependent current

The starting point of our numerical procedure is a *Monte Carlo* sampling of the set of random quantities entering the stochastic Liouville equation Supplementary Equation 4, for each individual electron-hole spin pair. The set accounts for local electron and hole hyperfine-field strengths of Gaussian distributions centered around zero, and a random direction for the vector connecting the electron and hole sites, i.e., a unit vector distributed uniformly in all directions. These random quantities form the Floquet Hamiltonian H_F through Supplementary Equations 3, 9, and Eq. 1 of the main text. To this set we add a randomly generated direction for the local axis of quantization, defining the triplet recombination through Supplementary Equation 33 of Section S1.6 (another uniformly distributed unit vector).

For the given set of random quantities, we calculate the four matrix elements $\langle\psi_{\alpha,0}|\Pi_S|\psi_{\alpha,0}\rangle$, $\alpha = T_+, T_0, T_-, S$, compute $\mathrm{tr}\tilde{\rho}_0$ from Eq. 3, and average the resulting $\mathrm{tr}\tilde{\rho}_0$ over the random configurations. This calculation is performed after truncating the infinite-dimensional Floquet

Hamiltonian H_F and the corresponding Hilbert space. The truncation we employ consists of restricting n and m in Eq. 1 to run from $-N_0$ to N_0, where N_0 is a suitably chosen integer. Thus, the truncated Hilbert space is $4(2N_0+1)$-dimensional, spanned by the dressed states $|\alpha, n\rangle$ with $-N_0 \le n \le N_0$. Consequently, the truncated Hamiltonian H'_F and the projection operator Π'_S are $4(2N_0+1) \times 4(2N_0+1)$ matrices.

The eigenvectors $|\psi'_{\alpha,0}\rangle$ found from the numerical solution of the truncated eigenvalue equation $H'_F|\psi'_{\alpha,n}\rangle = \varepsilon'_{\alpha,n}|\psi'_{\alpha,n}\rangle$ are used in Eq. 3 to calculate $\mathrm{tr}\tilde{\rho}_0$. The sampling procedure is repeated N_s times and the experimental observable $I(B_0, B_1)$ sought is determined as the average $\langle \mathrm{tr}\tilde{\rho}_0 \rangle$. This procedure also controls the choice of N_0 and N_s since the truncation size N_0 is set by inspecting the convergence with increasing N_0 of the truncated matrix element $\langle \psi'_{\alpha,0}|\Pi'_S|\psi'_{\alpha,0}\rangle$ entering into Eq. 3. We verify that, in the parameter space of interest, $N_0 = 4$, corresponding to a 36×36 Hamiltonian H'_F, ensures acceptable convergence. This convergence is also related to the fact that the above matrix element arises between the eigenvectors $|\psi'_{\alpha,0}\rangle$ possessing the largest components $\langle \beta, m|\psi'_{\alpha,0}\rangle$ at $m \sim 0$, while the components at $m \sim \pm N_0$, which are more sensitive to the truncation procedure, have a minimal contribution to $\langle \psi'_{\alpha,0}|\Pi'_S|\psi'_{\alpha,0}\rangle$. The number of sampling steps, N_s, is chosen from considerations of accuracy of the *Monte Carlo* averaging. We find that $N_s = 10^5$ provides satisfactory accuracy, within an error of less than 2% of the output.

The specific parameters used in the simulations are listed in Supplementary Table 1. The numerical values of the two combinations of rate constants governing the steady-state solution of the stochastic Liouville equation, $w_d \equiv r_d + r_T + 1/T_{\mathrm{sl}}$ and $k_r \equiv r_S - r_T$, are in good agreement with previous experimental results[5]. The rate constant δr_T describing the fine structure of the triplet recombination ensures the accurate reproducibility of the $g \approx 4$ resonance feature observed in the experiment (see Section S1.6). Characteristic magnitudes of hyperfine fields $b_{\mathrm{hf},e}$ and $b_{\mathrm{hf},h}$ are equivalent to the standard deviations $\Delta B^{\mathrm{D}}_{\mathrm{hyp},1}$, $\Delta B^{\mathrm{D}}_{\mathrm{hyp},2}$ inferred from the analysis of the power-broadening of experimental line shapes in Supplementary Note 4. A more accurate, multi-frequency power broadening analysis carried out for the same material[6] yields the parameter values listed in Supplementary Table 1.

Supplementary Note 1.6: Amendments to the conventional spin-pair model

The above elaborations are based on the conventional spin-pair model of spin-dependent processes in organic semiconductors, see, e.g., Boehme & Lips[2] and Mkhitaryan *et al.*[3] Small modifications to this model are needed to rationalize the observations made here. The $g \approx 4$ feature seen clearly in our experiment demonstrates a resonant transition between the triplet states T_+ and T_-. This single-photon transition with the magnetic quantum number changing by $\Delta m = 2$ is possible due to the mixing of the spin-pair states by dipolar interactions[7,8]. However, under conditions precluding thermal spin polarization and with the assumption of equal dissociation and recombination rates from all triplet states, the states T_+ and T_- are populated equally. In this case, none of the spin-pair state populations are affected by a resonant interconversion between T_+ and T_- and therefore this resonance cannot induce a change in the device current. This absence of a detectable resonance is confirmed by our simulations within the conventional spin-pair model described above.

The experimentally observed $g \approx 4$ resonance feature is reproduced within the spin-pair model following a modification of the triplet recombination rates. The modification stems from the following arguments. In the course of triplet recombination, a weakly coupled electron-hole spin pair recombines into a triplet exciton. The exciton created is a triplet with zero-field principal axes determined by its local molecular environment. The resulting triplet exciton wavefunctions are quite different for the different spin projection states defined relative to the system of local principal axes. Hence, it is natural to expect that the recombination into these different states can occur with different rates.

We now assume that the rate of triplet spin-pair recombination depends on the specific spin projection state relative to the system of principal axes. Thus, instead of the single recombination rate r_T, we employ three different constants $r_{\tilde{T}_+}$, $r_{\tilde{T}_0}$, $r_{\tilde{T}_-}$ for the rates of recombination from the three triplet states in the local principal-axes system $\tilde{T}_+$, $\tilde{T}_0$, and $\tilde{T}_-$. We further find that the experimentally observed resonance features are reproduced quite accurately by setting

$$r_{\tilde{T}_0} = r_T, \qquad r_{\tilde{T}_+} = r_T + \delta r_T, \qquad r_{\tilde{T}_-} = r_T - \delta r_T, \tag{33}$$

where $\delta r_T \approx 0.072 w_d$. Remarkably, this modification leaves the *average* recombination rates unchanged, so that the recombination rates from the triplet states T_+, T_0, and T_- average to the same value, r_T. Note that the states $\tilde{T}_+$, $\tilde{T}_0$, and $\tilde{T}_-$ are linear combinations of T_+, T_0, and T_-.

The modified triplet recombination is easily incorporated in Supplementary Equation 31 and subsequently in the resulting expression for the spin-dependent current, Eq. 3. We introduce the difference of the projection operators onto the local $\tilde{T}_+$ and $\tilde{T}_-$ states, $\Delta \mathrm{P} = |\tilde{T}_+\rangle\langle\tilde{T}_+| - |\tilde{T}_-\rangle\langle\tilde{T}_-|$. In the laboratory basis T_+, T_0, T_-, S, this operator has the matrix representation

$$\Delta \mathrm{P} = \begin{pmatrix} \cos\theta & \frac{1}{\sqrt{2}}\sin\theta\, e^{i\phi} & 0 & 0 \\ \frac{1}{\sqrt{2}}\sin\theta\, e^{-i\phi} & 0 & \frac{1}{\sqrt{2}}\sin\theta\, e^{i\phi} & 0 \\ 0 & \frac{1}{\sqrt{2}}\sin\theta\, e^{-i\phi} & -\cos\theta & 0 \\ 0 & 0 & 0 & 0 \end{pmatrix}, \tag{34}$$

where $\theta \in (0, \pi)$ and $\phi \in (0, 2\pi)$ are the spherical angles characterizing the (random) local principal axes system. The Floquet extension of this operator, $\Delta\Pi = \sum_n \big(|\tilde{T}_+, n\rangle\langle\tilde{T}_+, n| - |\tilde{T}_-, n\rangle\langle\tilde{T}_-, n|\big)$, has the matrix representation, $\langle\alpha, n|\Delta\Pi\,|\beta, m\rangle = \Delta \mathrm{P}_{\alpha\beta}\delta_{nm}$. With the rates modified by Supplementary Equation 33 we get a modified expression of Eq. 3,

$$\mathrm{tr}\tilde{\rho}_0 \simeq \frac{G}{4}\sum_\alpha \frac{1}{w_d + k_r\langle\psi_{\alpha,0}|\Pi_S|\psi_{\alpha,0}\rangle + \delta r_T\langle\psi_{\alpha,0}|\Delta\Pi|\psi_{\alpha,0}\rangle}. \tag{35}$$

According to this relation, the set of random quantities from which $\mathrm{tr}\tilde{\rho}_0$ is calculated and subsequently averaged as described in the previous section must be extended to include a pair of spherical angles specifying a system of local principal axes oriented uniformly in all directions. With such an extension and by utilizing the obvious truncation of $\Delta\Pi$, the numerical procedure for the evaluation of the spin-dependent current basically repeats the steps described in the previous section.

Supplementary Note 2: Characterization of polymer OLEDs

See Supplementary Figure 1 for information on device fabrication and characterization.

Supplementary Note 3: Current change measurement as a function of B_0 and RF-power $P \propto B_1^2$

The silicon wafer accommodating the monolithic EDMR-microwire/OLED pixel was placed on a chip holder while electrical connections were established by gold wires and indium dots. The chip holder was placed on top of an aluminum chassis. This holder was then placed in the center of a Helmholtz coil setup for the application of the static B_0 field controlled by a Kepco ATE100-10M constant-current source. An RF signal was generated by an Agilent N5181A frequency generator and amplified by an ENI 510L RF power amplifier (9.5 W linear, 45 dBm, 1.7-500 MHz), the output of which was directly connected to the microwire. The largest values of the amplitude B_1 of the RF radiation were generated across the narrowest segment of the microwire, right beneath the active device area. The device was biased using a Keithley 2400 source meter and the current was converted by a Stanford Research current preamplifier SRS570 (500 nA offset, 500 nA/V sensitivity with a 10 Hz low-pass filter) into a voltage fed into an NIPCI-6251DAQ analog/digital converter. Data acquisition took place using MATLAB. The steady-state device current was recorded for various values of B_0 with and without an 85 MHz RF field applied at different output powers P of the RF generator. Due to the amplifier employed and the non-critical coupling of amplifier and thin-film wire, an arbitrary but constant relationship $B_1 = c\sqrt{P}/2$ between P and B_1 exists as long as Ohmic heating effects, which affect the resistance of the Cu microwire, are negligible. The measurements were conducted at room temperature while the sample was operated in an inert atmosphere with a slow flow of N_2 gas. This conversion factor c was determined by considering the power broadening of the resonance spectra as described in Supplementary Note 4. The conversion factor was further corroborated using the Bloch-Siegert shift (BSS) as described in Supplementary Notes 5 through 7.

Supplementary Note 4: Determination of the $\sqrt{P}$ to B_1 conversion factor through power broadening

The conversion factor between applied power and B_1 was determined through measurements of power broadening (PB) of the EDMR resonance line at low driving powers. Supplementary Figure 2 displays a set of EDMR spectra measured for various applied RF powers. The data was fitted globally, i.e., the B_1-dependent resonance line shape was fitted to all data sets simultaneously, using three fit parameters: (i) the conversion factor $c_{\mathrm{PB}} = \frac{2B_1}{\sqrt{P}}$ and (ii) constant offsets determined by the intrinsic, hyperfine-field governed linewidths of the two carrier-pair resonances which define the resonance line width in the absence of power broadening. The precise numerical procedure used is described in detail elsewhere[9]. The results of this procedure are $c_{\mathrm{PB}} = 0.0217(60)$ $\mathrm{mT}/\sqrt{\mathrm{mW}}$ as well as $\Delta B^{\mathrm{D}}_{\mathrm{hyp},1} = 0.081(2)\ \mathrm{mT}$ for the narrow hole charge-carrier resonance and $\Delta B^{\mathrm{D}}_{\mathrm{hyp},2} = 0.28(20)\ \mathrm{mT}$ for the broad electron charge-carrier resonance line. Note that these values represent the standard deviation of the Gaussian spin-resonance spectra as defined in Joshi *et al.*[10] and Malissa *et al.*[11]; they do not represent the full width at half maximum (FWHM) of the charge-carrier spin-resonance spectral lines as discussed by Waters *et al.*[12], which are larger than the standard deviations by a factor of $2\sqrt{2\ln 2}$.

Supplementary Note 5: Hyperfine-field controlled linewidths of the spin-pair resonance

The widths of the two low-frequency (<1 GHz) low-power EDMR resonances in MEH-PPV are known to be determined by the expectation values of the two local hyperfine-field distributions $\Delta B^{\mathrm{D}}_{\mathrm{hyp},1}$and $\Delta B^{\mathrm{D}}_{\mathrm{hyp},2}$[6,9,10-12]. In fact, the widths of the two carrier resonance lines reported here compare to those reported previously for fully protonated h-MEH-PPV, $\Delta B^{\mathrm{H}}_{\mathrm{hyp},1} = 0.2080(8)$ mT for the narrow hole resonance and $\Delta B^{\mathrm{H}}_{\mathrm{hyp},2} = 0.8111(27)$ mT for the broad electron resonance[10,11], according to the ratios of $\frac{\Delta B^{\mathrm{D}}_{\mathrm{hyp},1}}{\Delta B^{\mathrm{H}}_{\mathrm{hyp},1}} = 0.389(9)$ and $\frac{\Delta B^{\mathrm{D}}_{\mathrm{hyp},2}}{\Delta B^{\mathrm{H}}_{\mathrm{hyp},2}} = 0.34(25)$. While the latter shows reasonable agreement with the ratio $\mu_{\mathrm{D}}/\mu_{\mathrm{H}} = 0.3070121$ of the nuclear magnetons of deuterium and protium, the former is slightly higher, likely because other inhomogeneous line-broadening effects may have influenced the narrow resonance line of the deuterated material under these conditions of very low hyperfine fields.

Supplementary Note 6: Determination of the $\sqrt{P}$ to B_1 conversion factor by the Bloch-Siegert shift

The detection of the spin-Dicke effect can be used in order to observe another signature of ultra-strong coupling, namely the breakdown of the rotating-wave approximation for the description of light-matter interaction. The physical origin of the latter, which expresses itself by a shift of the resonance frequency with increasing drive strength, the Bloch-Siegert shift (BSS)[13], is illustrated in Supplementary Figure 3. The graph shows plots of the transition probability

$$w_{\downarrow\to\uparrow}(B_0) = \overline{|\langle\psi(t)|\uparrow\rangle|^2} = \frac{\gamma B_1}{2\Omega_{\pm}(B_0)}, \tag{36}$$

representing the time average of Rabi's formula[13,14] from the ground state $|\downarrow\rangle$ of a $s = ½$ electron spin to the excited state $|\uparrow\rangle$ as a function of an applied static magnetic field B_0 for the conditions of ultrastrong and weak resonant drive. The red line indicates right-handed helicity of the RF

radiation, generating a Rabi frequency Ω_{+}, with blue labelling the left-handed helicity generating a Rabi frequency Ω_{-}. γ = 28.03 GHz/T is the electron spin's gyromagnetic ratio, which is related to the g-factor through $\gamma = g\mu_B/\hbar$, and

$$\Omega_{\pm}(B_0) = \sqrt{(\gamma B_1)^2 + (\gamma B_0 \mp \omega)^2} \tag{37}$$

are the Rabi frequencies of the two helicity components with $\omega = 2\pi f$ being the frequency of the applied radiation, assuming that there is no spin-orbit coupling, local random hyperfine interactions, coupling to other spin systems, or coupling that could cause spin relaxation. The plots in Supplementary Figure 3 are based on RF driving fields with f = 85 MHz, and B_1 = 3.5 mT and 0.5 mT radiation for the strong and weak-drive cases, respectively. B_1 is linearly polarized in an $\hat{x}$-$\hat{y}$ plane, while B_0 is parallel to $\hat{z}$. Supplementary Figure 3 also displays normalized sums of the blue and red plots (black lines), i.e., superpositions of the two helicities, describing the case of linearly polarized RF radiation.

When the inhomogeneous broadening due to, e.g., the hyperfine-field distribution, is small compared to B_1, the line shape described by Supplementary Equation 34 will be predominantly Lorentzian with a full-width-at-half-maximum (FWHM) of $2B_1$, an effect referred to as power broadening. When $B_1 \ll B_0$, distinct narrow resonance lines emerge whose centers and shapes are nearly equal for linear and circular polarization states, even though for the circularly polarized excitation, the resonance peaks occur only when the field direction is positive for the right-handed helicity and negative for the left-handed helicity, i.e., for the EPR-active helicities as shown in Supplementary Figure 3. This behavior is explained by the rotating-wave picture where the Rabi precession of the electron spin nutates due to a constant magnetic field B_1 along the $\hat{x}$-axis in a reference frame which rotates along with the EPR-active helicity of the circularly polarized light when the spin's Larmor frequency $\omega_0 = \gamma B_0$ and the radiation frequency ω are identical, i.e., in resonance. No such nutation can take place for the EPR-inactive helicity since $\omega_0 \neq -\omega$. The rotating-frame picture is not only illustrative, its mathematical implementation in the form of the rotating-wave approximation also allows the time-dependent perturbation by circularly polarized light to render the spin-Hamiltonian an exactly solvable problem. Under application of linear polarization, resonance peaks occur in both positive and negative field directions as the linear

polarization state is the superposition of the two circular polarization states. Under weak drive, the resonance peaks for both positive and negative static field values shown in Supplementary Figure 3 are identical, and thus linearly polarized excitation can be approximated by a rotating wave. This approximation is equivalent to circularly polarized excitation with positive helicity, at positive static fields, under complete neglect of the out-of-resonance helicity.

Under ultrastrong drive, power broadening becomes large enough such that the contributions of the EPR-active and EPR-inactive helicities superimpose. The rotating-wave approximation then breaks down, causing a BSS of the local frequency maximum (the peak center frequency) $\delta\omega_0 = \frac{(\gamma B_1)^2}{4\omega_0}$ relative to the Larmor frequency[15,16]. The positive BSS of the resonance frequency with increasing B_1 causes a negative BSS of the center magnetic field B_c

$$\delta_{BSS} = \frac{B_c}{2} - \sqrt{\left(\frac{B_c}{2}\right)^2 - \left(\frac{B_1}{2}\right)^2} \tag{38}$$

under experimental conditions where a constant drive-field frequency f is applied and the magnetic field is swept as seen in the illustration in Supplementary Figure 3 for the broad resonance lines. Detection of the BSS through the shift of the center of the magnetic resonance line is therefore possible, but only within the ultrastrong drive regime (as the BSS is too small when $B_1 \ll B_0$). Furthermore, in the regime of the so-called deep-strong drive where $B_1 > B_0$, the resonance maximum will become pinned at $B_0 = 0$. Within the intermediate regime of strong drive, the BSS becomes progressively more pronounced, while the resonance peak becomes smaller due to the effect of power broadening (cf. Supplementary Figure 2). An accurate determination of the peak center as needed to quantify the BSS then becomes difficult with conventional magnetic resonance spectroscopy.

It is the particular signature of spin-collectivity in the device current on resonance, the sign reversal of spin-dependent current changes under strong drive conditions, which allows us to circumvent this problem arising from power broadening. Since the spin-Dicke effect inverts ΔI on resonance only, i.e., within a narrow range $|\omega - \gamma B_0| \lesssim \mu_B B_{\text{hyp}}/\hbar$ around the resonance condition, it is not subject to power broadening. Thus, while power broadening increases with B_1, the spin-Dicke

effect remains an indicator for the resonance peak center with an accuracy that is effectively independent of B_1. Indeed, the data in Fig. 3 of the main text shows that the width of the inverted current change $\Delta I(B_0)$ due to the spin-Dicke effect [feature (i) in Fig. 3 of the main text] remains almost unchanged as B_1 is increased, in contrast to the non-inverted conventional carrier-pair spin resonance, which shows strong power broadening. The current changes measured at the highest driving powers corresponding to $B_1 > 2$ mT show such strong power broadening—the blue regions in Fig. 3 represent current quenching due to very broad EDMR peaks—that the peak center cannot be determined with sufficient accuracy to corroborate the BSS. Only with the spin-Dicke effect as observed in Fig. 3 [feature (i)] a clear shift of the resonance center of the red area in the vertical (B_0) direction towards lower values of B_0 can be identified.

Supplementary Figure 4a displays several selected experimental magnetoresistance data sets $\Delta I(B_0)$ extracted from the data of Fig. 3 of the main text for various values of P. In order to exploit the quantitative nature of the BSS for the determination of the $\sqrt{P}$ to B_1 conversion, all extrema for all spectra were determined as a function of $\sqrt{P}$ using a standardized procedure. To find the center values of magnetic field B_0^{c} of the $g \approx 2$ resonance for each applied RF power P, the data for each spectrum $\Delta I(B_0)$ was subjected to a 40 data-point smoothing procedure using the *Origin* Software *FFT smoothing method* (https://www.originlab.com/doc/Origin-Help/Smoothing). Subsequently, the package's peak analyzer was used to find the absolute maximum defining B_0^{c} for each value of P. The resulting dependency of $B_0^{\mathrm{c}}(\sqrt{P})$ shown in Supplementary Figure 4b was then fitted with the expression

$$B_0^{\mathrm{c}}(\sqrt{P}) = \frac{B_0^{\mathrm{c}}(0)}{2} \pm \sqrt{\left[\left(\frac{B_0^{\mathrm{c}}(0)}{2}\right)^2 - \left(\frac{B_1}{2}\right)^2\right]} \tag{39}$$

in which $B_1 = \frac{c_{\mathrm{BSS}}\sqrt{P}}{2}$ and $B_0^{\mathrm{c}}(0) = f/\gamma = 3.0325$ mT was defined by the gyromagnetic ratio $\gamma = 28.03\,\frac{\mathrm{GHz}}{\mathrm{T}}$ and the applied radiation frequency $f = 85$ MHz. The fit was therefore reduced to a single fit variable, namely the conversion factor $c_{BSS} = \frac{2B_1}{\sqrt{P}} = 0.018(2)\mathrm{mT}/\sqrt{\mathrm{mW}}$.

The standard deviation (the error) for B_0^{c} was estimated on the basis of the residuals of the set of peak values $B_0^{\mathrm{c}}(\sqrt{P})$ obtained by the procedure described above and the fit results based on Supplementary Equation 37. The distribution of these residuals is shown in the histogram in Supplementary Figure 5. Using this distribution and an estimator for an unbiased sample variance, a standard deviation of 0.106 mT was found for B_0^{c}. We note that the center of this distribution has a slight offset of -0.0417 mT, which is due to an emphasis of the residuals of the fit function for powers in the range of 40 $\sqrt{\mathrm{mW}} \leq \sqrt{P} \leq$ 100 $\sqrt{\mathrm{mW}}$. This offset is caused by the fact that the fit model does not account for the half-field resonance signal (red arrow in Supplementary Figure 4), affecting the precision in determining B_0^{c}.

Supplementary Note 7: Spin-Dicke effect of perdeuterated d-MEH-PPV

Supplementary Figure 6 displays the plot of the on-resonance ($g \approx 2$) current change normalized to its minimum as a function of the driving-field amplitude B_1, and scaled along the abscissa to the local hyperfine-field strength B_{hyp} experienced by the charge carrier for fully protonated (black), perdeuterated (red), and partially deuterated[12] (blue) MEH-PPV. The increase of this function after an initial linear decrease is characteristic for the onset of the spin-Dicke effect[17], and these functions are predicted to solely scale with B_{hyp}. For the plots shown in Supplementary Figure 6, the scaling factors of $B_{\mathrm{hyp}}^{\mathrm{H}} = 0.97$ mT and $B_{\mathrm{hyp}}^{\mathrm{DH}} = 0.51$ mT for the fully protonated and mixed MEH-PPV were taken from Waters *et al.*[12], while the scaling factor $B_{\mathrm{hyp}}^{\mathrm{D}} = 0.24(3)$ mT for perdeuterated MEH-PPV was obtained from the data presented here as discussed above in Supplementary Note 5. This value was obtained through adjustment of the scaling factor such that maximal overlap with the other two functions occurred under the assumption that the scale for B_1 is given by the power conversion factor c_{BSS} as obtained from the fit of the BSS discussed in Supplementary Note 6. This procedure leads to a ratio $B_{\mathrm{hyp}}^{\mathrm{D}}/B_{\mathrm{hyp}}^{\mathrm{H}}$ = 0.25(3) of the scaling factors, which is slightly smaller than the ratio $\mu_{\mathrm{D}}/\mu_{\mathrm{H}}$ = 0.3070121 of the nuclear magnetons of deuterium and protium.

The experiments on perdeuterated MEH-PPV reported here provide significantly improved ratios of B_1/B_{hyp} in comparison to earlier work[9,12]. The observation made under these conditions confirms the theoretically predicted saturation behavior of the current change in the spin-Dicke regime beyond the onset of the spin-Dicke effect, i.e., where the sign change of ΔI occurs[17]. The gradual decrease of the change in saturation current with increasing B_1 is a consequence of the BSS, which causes the resonance to detune from the $g \approx 2$ center magnetic field at high driving powers.

Supplementary Figures

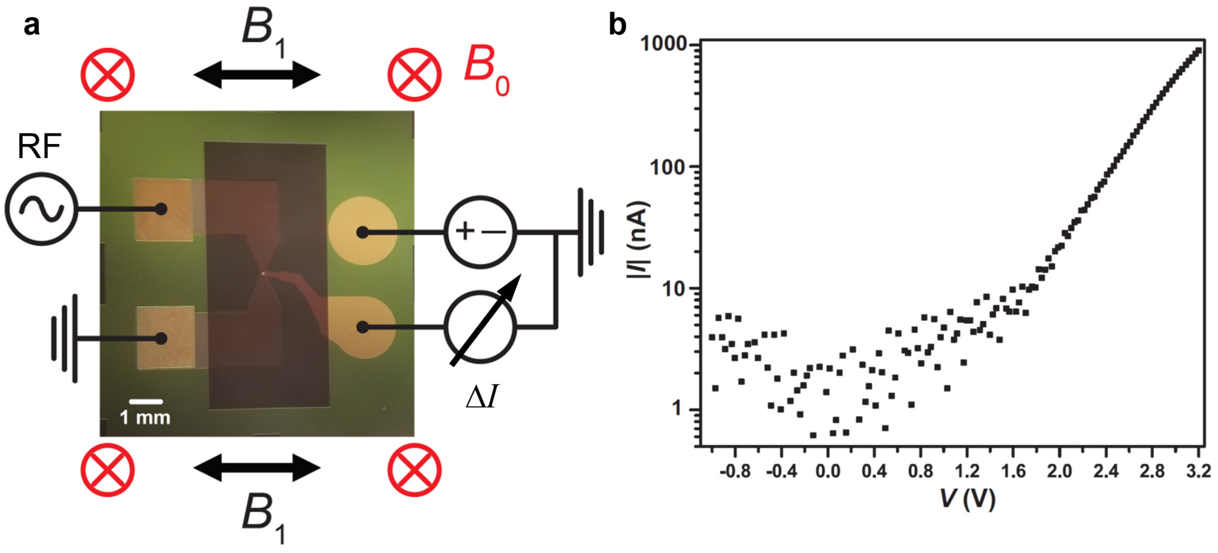

Supplementary Figure 1. Device structure and characterization. a, *Photograph of a high-B_1 monolithic thin-film wire/OLED device with diagrams illustrating the electric circuitry used for the RF excitation as well as the measurement of the device current. The RF field polarization generated by the thin-film wire of amplitude B_1 (black arrows) is oriented within the sample plane. The externally applied static magnetic field B_0 is oriented perpendicular to the sample plane. The active OLED pixel of diameter 57 µm is seen as the bright spot at the center of the image.* **b,** *Current-voltage (I-V) characteristics of the micronscale OLED device.*

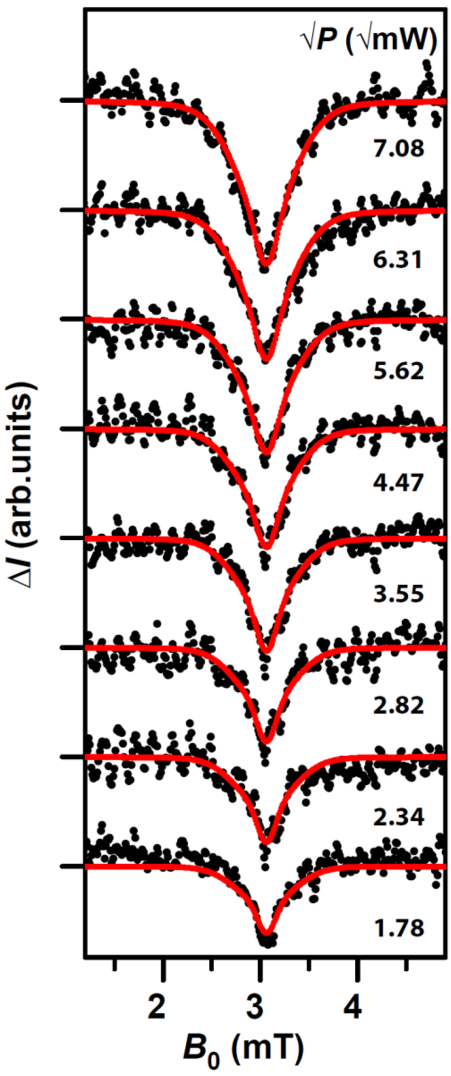

Supplementary Figure 2. Power dependence of EDMR spectra. *Black dots: plots of OLED current change* Δ*I as a function of the applied magnetic field* B_0 *in the presence of an 85 MHz RF radiation field at various applied RF powers as indicated for each data set. Red lines: simultaneous global fits of all experimental data sets using a model in which the resonance line width is governed by power broadening and the intrinsic Gaussian hyperfine distributions experienced by electron and hole spins.*

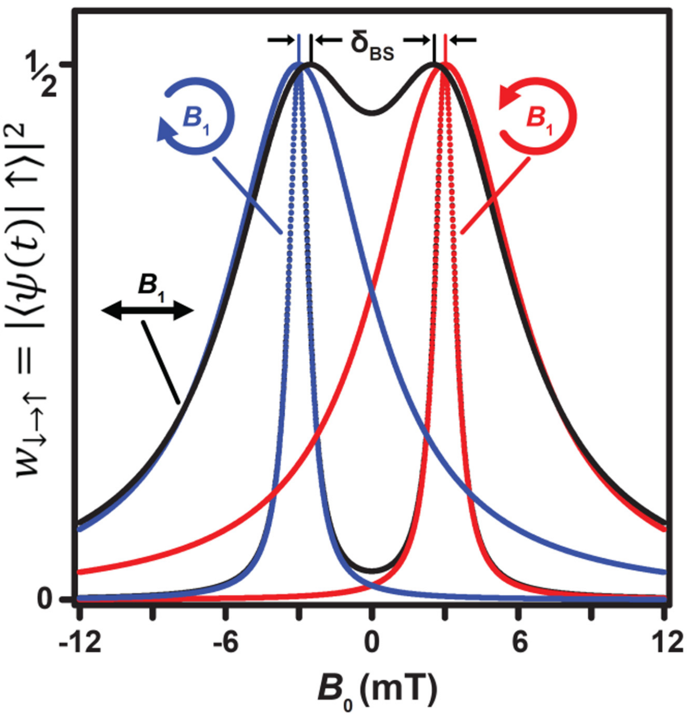

Supplementary Figure 3. Origin of the Bloch-Siegert shift. *Illustration of the spin transition probability changes induced by magnetic resonance for weak (narrow peaks) and strong (broad peaks) electromagnetic drive conditions under linearly polarized excitation (black lines) and under circularly polarized excitation (red, blue). The simulated line shapes are normalized.*

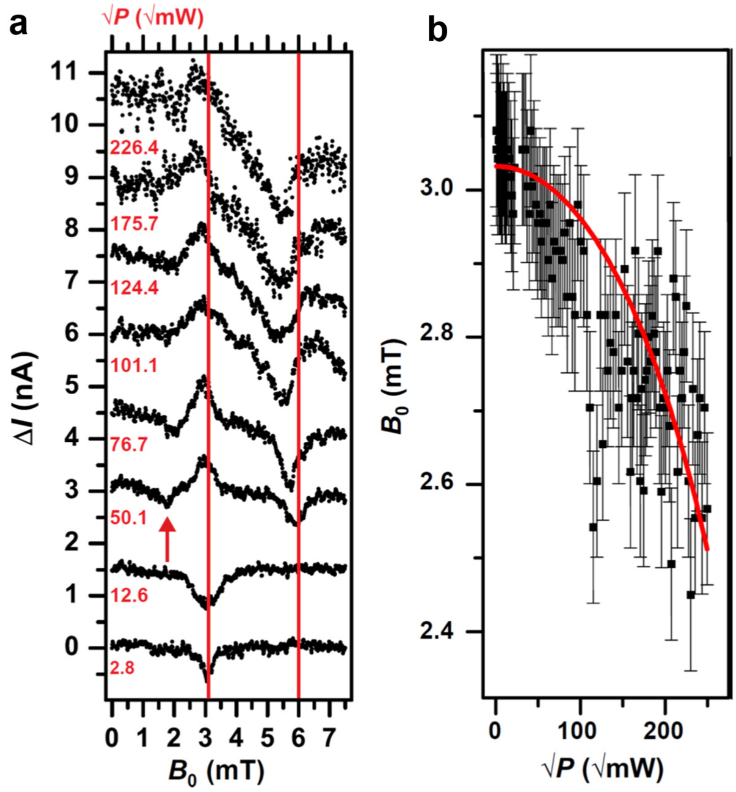

Supplementary Figure 4. Bloch-Siegert shift in OLED EDMR. a, *Plot of the current change ΔI as a function of the static magnetic field B_0 in the presence of RF radiation with different applied powers as indicated for each data set (red labels), corresponding to different driving-field amplitudes B_1. The red lines indicate the resonance centers of the $g \approx 2$ and the $g \approx 1$ lines for small B_1. The arrow indicates the half-field resonance.* **b,** *Black points: plot of the resonance-peak extrema of the data shown in panel* **a** *as a function of the applied driving field amplitude $B_1 = c\sqrt{P}$. Red line: fit of the data using a model function based on the BSS.*

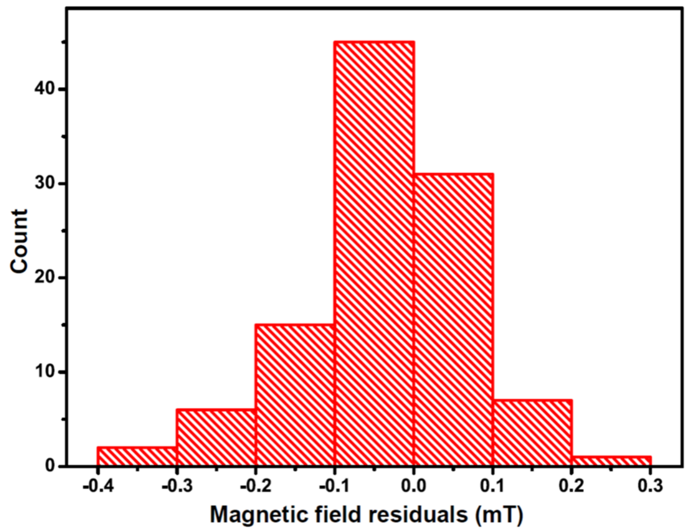

Supplementary Figure 5. Fitting residuals. *Histogram showing the number of counts for the fit residuals of the magnetic-field peak center value* B_0^c *from fitting to Supplementary Equation 37.*

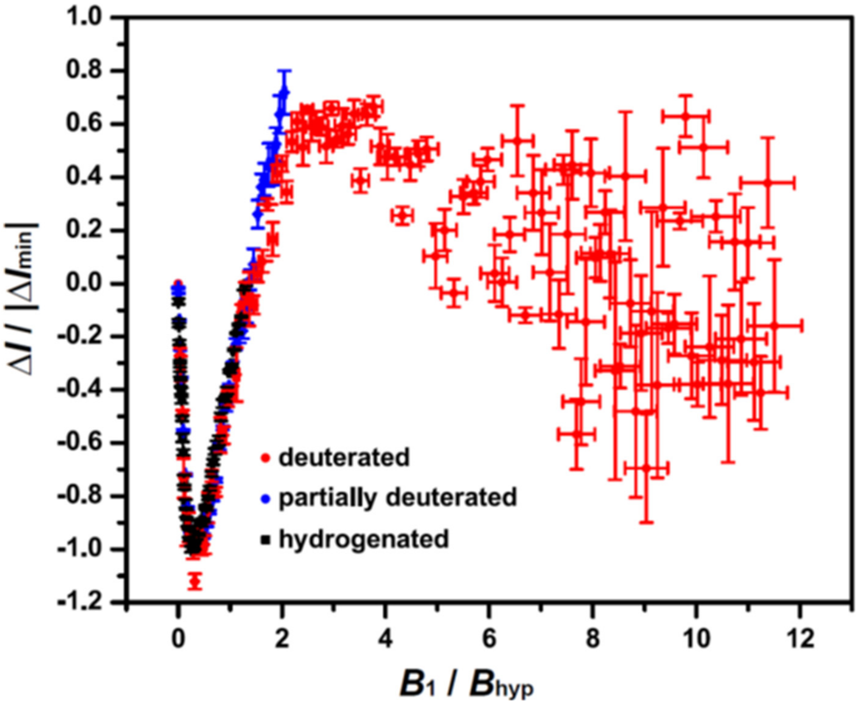

Supplementary Figure 6. Comparison of EDMR amplitudes for three different materials. *Plot of the on-resonance current change (B_0 = 3.05 mT, f = 85 MHz) normalized to the current change when the current minimum occurs, as a function of the amplitude of the driving field B_1 normalized to the expectation value of hyperfine field strength for three different OLED samples. The three data sets correspond to devices with hydrogenated, partially deuterated (both taken from Waters* et al.[12]*) and fully deuterated MEH-PPV.*

Supplementary Tables

Supplementary Table 1: *Parameter values used in the simulations of spin-dependent current.*

w_d(kHz)	k_r(kHz)	δr_T(kHz)	$b_{\mathrm{hf},e}$(mT)	$b_{\mathrm{hf},h}$(mT)	D(mT)	J(mT)
290.1	58.8	21	0.076	0.244	0.22	0.065

Supplementary References

1. van Schooten, K. J., Baird, D. L., Limes, M. E., Lupton, J. M. & Boehme, C. Probing long-range carrier-pair spin-spin interactions in a conjugated polymer by detuning of electrically detected spin beating. *Nat. Commun.* **6**, 6688 (2015).
2. Boehme, C. & Lips, K. Theory of time-domain measurements of spin-dependent recombination with pulsed electrically detected magnetic resonance. *Phys. Rev. B* **68**, 245101 (2003).
3. Mkhitaryan, V. V., Danilović, D., Hippola, C., Raikh, M. E. & Shinar, J. Comparative analysis of magnetic resonance in the polaron pair recombination and the triplet exciton-polaron quenching models. *Phys. Rev. B* **97**, 035402 (2018).
4. Shirley, J. H. Solution of the Schrödinger Equation with a Hamiltonian Periodic in Time. *Phys. Rev.* **138**, B979-B987 (1965).
5. Baker, W. J., Keevers, T. L., Lupton, J. M., McCamey, D. R. & Boehme, C. Slow Hopping and Spin Dephasing of Coulombically Bound Polaron Pairs in an Organic Semiconductor at Room Temperature. *Phys. Rev. Lett.* **108**, 267601 (2012).
6. Stoltzfus, D. M. *et al.* Perdeuteration of poly[2-methoxy-5-(2′-ethylhexyloxy)-1,4-phenylenevinylene] (d-MEH-PPV): control of microscopic charge-carrier spin–spin coupling and of magnetic-field effects in optoelectronic devices. *J. Mater. Chem. C* **8**, 2764-2771 (2020).
7. Schweiger, A. & Jeschke, G. *Principles of Pulse Electron Paramagnetic Resonance*, Oxford University Press (2001).
8. Gromov, I. & Schweiger, A. Multiphoton Resonances in Pulse EPR. *J. Magn. Reson.* **146**, 110-121 (2000).
9. Jamali, S., Joshi, G., Malissa, H., Lupton, J. M. & Boehme, C. Monolithic OLED-Microwire Devices for Ultrastrong Magnetic Resonant Excitation. *Nano Lett.* **17**, 4648-4653 (2017).
10. Joshi, G. *et al*. Separating hyperfine from spin-orbit interactions in organic semiconductors by multi-ocatve magnetic resonance using coplanar waveguide microresonators. *Appl. Phys. Lett.* **109**, 103303 (2016).

11. Malissa, H. *et al*. Revealing weak spin-orbit coupling effects on charge carriers in a π-conjugated polymer. *Phys. Rev. B* **97**, 161201(R) (2018).
12. Waters, D. P. *et al*. The spin-Dicke effect in OLED magnetoresistance. *Nat. Phys.* **11**, 910-914 (2015).
13. Bloch, F. & Siegert, A. Magnetic Resonance for Nonrotating Fields. *Phys. Rev.* **57**, 522-527 (1940).
14. Gross, M. & Haroche, S. Superradiance: An essay on the theory of collective spontaneous emission. *Phys. Rep.* **93**, 301-396 (1982).
15. Malissa, H. *et al*. Room-temperature coupling between electrical current and nuclear spins in OLEDs. *Science* **345**, 1487-1490 (2014).
16. Wei, C., Windsor, A. S. M. & Manson, N. B. A strongly driven two-level atom revisited: Bloch-Siegert shift versus dynamic Stark splitting. *J. Phys. B: At. Mol. Opt. Phys.* **30**, 4877-4888 (1997).
17. Roundy, R. C. & Raikh, M. E. Organic magnetoresistance under resonant ac drive. *Phys. Rev. B* **88**, 125206 (2013).
